# High- and Moderate-Risk Variants Among Breast Cancer Patients and Healthy Donors Enrolled in Multigene Panel Testing in a Population of Central Russia

**DOI:** 10.3390/ijms252312640

**Published:** 2024-11-25

**Authors:** Syuykum Shumilova, Anastasia Danishevich, Sergey Nikolaev, George Krasnov, Anna Ikonnikova, Darya Isaeva, Sergei Surzhikov, Alexander Zasedatelev, Natalia Bodunova, Tatiana Nasedkina

**Affiliations:** 1 Engelhardt Institute of Molecular Biology, the Russian Academy of Sciences, 119991 Moscow, Russia; sofnya@gmail.com (S.S.); gskrasnov@mail.ru (G.K.); anyuik@gmail.com (A.I.); ssergey77@mail.ru (S.S.); zas@biochip.ru (A.Z.); 2 SBHI Moscow Clinical Scientific Center named after Loginov of Moscow Healthcare Department, 111123 Moscow, Russia; a.danishevich@mknc.ru (A.D.); s.nikolaev@mknc.ru (S.N.); d.isaeva@mknc.ru (D.I.); n.bodunova@mknc.ru (N.B.)

**Keywords:** breast cancer risk, multigene panel testing, pathogenic variant, *BRCA1/2*, *ATM*, patients, healthy controls

## Abstract

Assessments of breast cancer (BC) risk in carriers of pathogenic variants identified by gene panel testing in different populations are highly in demand worldwide. We performed target sequencing of 78 genes involved in DNA repair in 860 females with BC and 520 age- and family history-matched controls from Central Russia. Among BC patients, 562/860 (65.3%) were aged 50 years or less at the time of diagnosis. In total, 190/860 (22%) BC patients were carriers of 198 pathogenic/likely pathogenic (P/LP) variants in 30 genes, while among controls, 32/520 (6.2%) carriers of P/LP variants in 17 genes were identified. The odds ratio [95% confidence interval] was 16.3 [4.0–66.7] for *BRCA1*; 12.0 [2.9–45.9] for *BRCA2*; and 7.3 [0.9–56.7] for *ATM* (*p* < 0.05). Previously undescribed *BRCA1/2, ATM,* and *PALB2* variants, as well as novel recurrent mutations, were identified. The contribution to BC susceptibility of truncating variants in the genes *BARD1*, *RAD50*, *RAD51C*, *NBEAL1* (p. E1155*), and *XRCC2* (p. P32fs) was evaluated. The *BLM*, *NBN*, and *MUTYH* genes did not demonstrate associations with BC risk. Finding deleterious mutations in BC patients is important for diagnosis and management; in controls, it opens up the possibility of prevention and early diagnostics.

## 1. Introduction

Breast cancer retains first place among all cancers in women and thus represents a global public health problem, leading to a significant reduction in quality of life and mortality worldwide [1]. The study of genetic causes of BC and ovarian cancer (OC) development has revealed a number of genes whose mutations are associated with hereditary breast and ovarian cancer syndrome, meaning increased predisposition to these types of cancer. The *BRCA1/BRCA2* genes, which are the main target of genetic testing, should be mentioned first, as well as the *PALB2*, *CHEK2*, and *ATM* genes [2,3,4].

On the other hand, deleterious mutations in the above-mentioned genes explain only about 20% of all cases of familial breast cancer. In recent years, multigene panels have been actively used not only in the genetic testing of patients for accurate diagnosis of diseases but also in medical and genetic counseling [5,6,7,8,9]. This approach allows simultaneous analysis of pathogenic variants in several genes that may be responsible for inherited cancer syndromes. The multigene panels can also detect genetic variants in poorly studied gene regions, including non-coding intronic regions, and thus may result in the identification of previously unrecognized genetic determinants contributing to cancer susceptibility [10,11,12].

As BC susceptibility testing using gene panels becomes more and more accessible to patients, data are rapidly accumulating and being used in medical and genetic counseling. A number of studies conducted in clinical laboratories at medical centers or in commercial laboratories have demonstrated the effectiveness of detecting clinically significant variants compared to testing *BRCA1/2* genes alone. The increase in the number of clinically relevant findings can range from 5 to 10% depending on the study design, including the number of genes tested and patient inclusion criteria [13,14,15,16]. In most of these studies, BC patients were considered to be at high risk for hereditary BC (triple-negative cancer, or a family history of BC or OC). [17,18]. In the study of 77,085 women with BC using gene panel testing on the base of four large clinical laboratories in the USA, 24.1% of patients were found to be carriers of clinically significant genetic variants compared to 7.8% of BC patients tested according to current guidelines [19].

There is much less evidence to conclude about the risk of BC in women not selected by family history, which is important to consider in population-based screening. Large case-control studies were performed using different gene sets, for example, eight cancer predisposition genes excluding *BRCA1/2* genes, to analyze the contribution of moderate risk genes such as *ATM*, *PALB2*, *CHEK2*, and so on [20]. Other large investigations were carried out. The CARRIERS consortium study is based on the multigene panel testing of 28 genes and included more than 32,000 patients and 32,000 healthy women from the United States of America [21]. In addition, the International Study (BRIDGES), which included 60,000 women with breast cancer and more than 53,000 women without breast cancer, tested 34 putative susceptibility genes [22]. These studies have provided generalized cancer risk scores associated with pathogenic variants in known breast cancer predisposition genes to guide genetic counseling. On the other hand, estimates of breast cancer risk in carriers of pathogenic variants in selected populations are equally important. An example is a large case-control study of 1464 women diagnosed with breast cancer and 862 age-matched controls participating in the Australian Breast Cancer Family Study, and 6549 healthy, older Australian women enrolled in the ASPirin in Reducing Events in the Elderly (ASPREE) study for rare germline variants using a 24-gene panel [23].

In comparison to other European populations, Slavic populations are characterized by a high percent of founder mutations, primarily in the *BRCA1/2* genes, which are largely distributed in Poland, Belarus, Ukraine, and Russia [24], but also in other cancer susceptibility genes [25]. The Russian Federation has a long-standing practice of genetic testing for a limited set of underlying mutations [26,27]. NGS studies of BC patients with a negative result when tested for founder mutations allowed to identify a significant number of new variants, primarily in the *BRCA1/2* genes [28]. In addition, due to the extremely complex and heterogeneous ethnic composition of Russian Federation residents, testing the populations in different geographical areas is of great interest, as it provides an opportunity to obtain new data on the role of different variants, both in *BRCA1/2* and other genes involved in the development of the disease [29,30,31].

Here, we present data on the prevalence and risk assessment of BC associated with clinically significant variants in known susceptibility genes (*BRCA1*, *BRCA2*, *ATM*, *CHEK2*, and *PALB2*), as well as in disputed genes with an undetermined contribution to the disease (*BLM*, *NBN*, *RAD50*, *NBEAL1*, *XRCC2*, *MUTYH*, *FANCC*, and so on), identified in a panel of 78 genes involved in DNA repair processes. This study was conducted among breast cancer patients living in the regions of Central Russia (with an emphasis on those with an early age at disease onset) with comparison to a control group of healthy donors matched in age and family history with respect to cancer.

## 2. Results

### 2.1. Characteristics of Patients with Breast Cancer and Healthy Controls

A total of 860 female patients with BC and 520 healthy females who received genetic consultation in the Department of Personalized Medicine of The Loginov Moscow Clinical Scientific Center from 2019 to 2022 were included in this study. All patients had histologically confirmed breast cancer. The average age was 48 years (ranging from 18 to 83) for BC patients and 51 years (ranging from 24 to 87) for controls. Molecular classification of breast cancer was available in 781 cases. Among these, the luminal A subtype was found in 193 patients (25%), luminal B in 374 (48%), Her2-positive BC in 47 (6%), and triple-negative BC in 167 patients (21%). A total of 69/860 (8.0%) had another tumor, among which 38/860 (4.4%) patients had bilateral BC, and in 34/860 (4.0%) cases, BC was combined with other cancers. Three patients (3/860 or 0.4%) had bilateral BC in combination with other non-BC tumors.

The family history of cancer was provided for 843 patients with BC and 520 healthy controls. A total of 360/843(43%) patients and 236/520 (45%) healthy controls had relatives diagnosed with cancer. There was a positive family history of BC or ovarian cancer in 178/843 (21%) patients and 94/520 (18%) healthy controls, respectively. First-degree relatives with BC or ovarian cancer were present in 95/843 (12%) cases with BC and in 68/520 (13%) control cases. The detailed characteristics of the cohorts are given in Table 1.

### 2.2. Multigene Panel Sequencing

**Figure 1:**
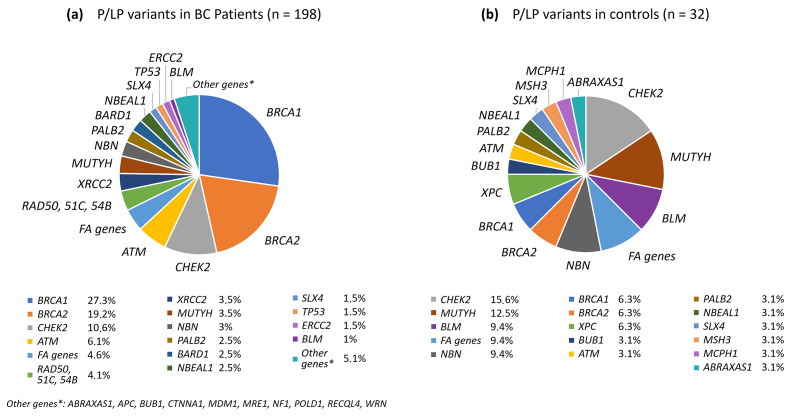
Distribution of P/LP variants in different genes in BC patient carriers (**a**) and healthy control carriers (**b**). The lower part of the figure shows a ranking of genes by decreasing frequencies of mutations.

There were 860 patients with BC and 520 healthy controls tested with a multigene panel, including the coding regions of 78 genes (Table S1). The full list of pathogenic (P) or likely pathogenic (LP) variants is given in Tables S2 and S3. The distribution of P/LP variants in BC patient carriers and healthy control carriers is in Figure 1.

In total, 198 P/LP variants in 30 genes among 190/860 (22%) BC patients and 32 P/LP variants in 17 genes among 32/520 (6.2%) healthy controls were identified, with an odds ratio (OR) = 4.3 [95% CI: 2.9–6.4], *p* < 0.0001 (to calculate the OR, the number of variant carriers was taken into account). Eight BC patients simultaneously carried two P/LP variants. If we considered only P/LP variants leading to protein chain truncation, the number of such variants was 155 in 151/860 (17.6%) BC patients and 23 variants in 23/520 (4.4%) controls, OR = 4.6 [95% CI: 2.9–7.2], *p* < 0.0001. Among BC patients, the *BRCA1*, *BRCA2*, *CHEK2*, *ATM*, *XRCC2*, *MUTYH*, *NBN*, *PALB2*, *BARD1*, *RAD50*, *FANCC*, and *NBEAL1* genes were the most frequently mutated. Among controls, the *CHEK2*, *MUTYH*, *BLM*, *NBN*, *BRCA1*, and *BRCA2* genes were more often affected.

### 2.3. Estimation of Breast Cancer Risk

To estimate the association between germline variants found and breast cancer risk for different genes, ORs were calculated (Table 2).

**Figure 2:**
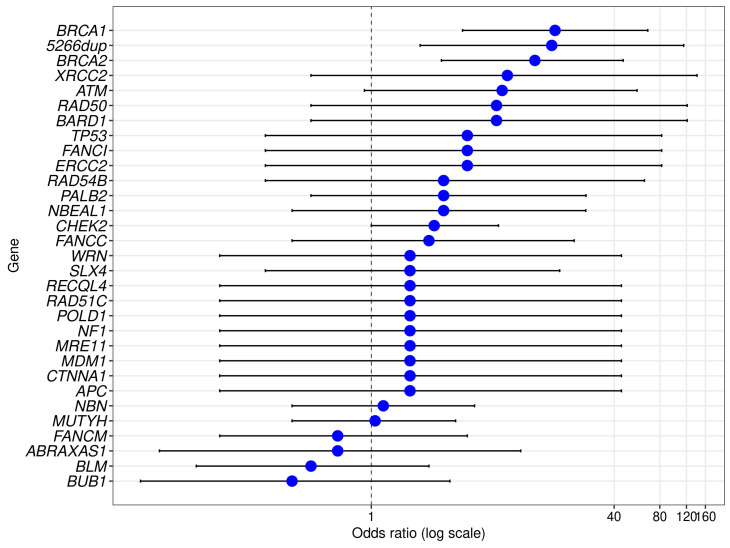
P/LP variants and breast cancer risk. ORs (large dots) and corresponding 95% confidence intervals (horizontal lines) for the association between breast cancer and pathogenic variants in various genes, sorted by OR.

We found evidence of a statistically significant association with breast cancer risk for three genes, with ORs of 16.3 [95% CI: 4.0–66.7] (*p* = 0.0001) for *BRCA1*, 12.0 [95% CI: 2.9–45.9] for *BRCA2* (*p* = 0.0001), and 7.3 [95% CI: 0.9–56.7] (*p* = 0.037) for *ATM* (Table 2, Figure 2).

### 2.4. Spectrum and Frequency of P/LP Variants in BRCA1 and BRCA2 Genes

A total of 96 (96/198 or 48.5%) P/LP variants or mutations in BC patients and healthy controls were detected in the *BRCA1/2* genes, with 56 mutations in *BRCA1* and 40 in *BRCA2*. Among the *BRCA1* gene mutations, 71% (40/56) were founder mutations previously described for Slavic populations in Russia, while 30% (16/56) can be attributed to non-founder mutations (Table 3). In so-called non-founder mutations, there were three recurrent variants, each occurring in two unrelated patients: c.4675G>C, c.4689C>G, and c.1303_1309del. One LP variant, c.4507del (p.Ser1503HisfsTer2), was not previously described. This variant was found in a 37-year-old woman with triple-negative BC and a family history of BC (her sister and grandmother also had BC).

**Figure 3:**
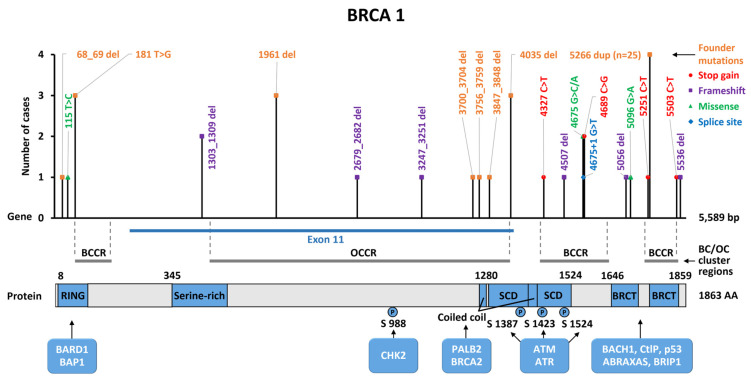
Location of P/LP variants found in the study along the *BRCA1* gene. Domain structure of the *BRCA1* gene: N-terminal RING (really interesting new gene) domain, serine-rich region, coiled-coil domain, SCD (serine-containing domain), and BRCT (BRCA1 C-terminal) repeats. BARD1, BAP1, CHEK2, PALB2, BRCA2, ATM, ATR, BACH1, CtIP, p53, ABRAXAS1, and BRIP1 bind to the BRCA1 protein at specific sites (domains and proteins binding to them are marked in blue).

The spectrum of P/LP variants in the *BRCA1* gene included 22 different types of nucleotide substitutions, of which 77% (17/22) were truncating mutations, 18% (4/22) were missense, and 5% (1/22) were mutations affecting splicing (Table S2, Figure 2). In total, 5/56 (9%) of P/LP variants were located in the RING domain, 13/56 (23%) were in exon 11, and 38/56 (68%) variants were found in the C-terminal part of the *BRCA1* gene (SCD and BRCT domains). Moreover, identified *BRCA1* P/LP variants were more frequently localized in so-called BC cluster regions compared to OS cluster regions (13/56 or 24% vs. 43/56 or 76%, *p* < 0.0001) (Figure 3). Overall, only 16/56 P/LP variants (29%) in the *BRCA1* gene were single, while 40/56 (71%) were represented by founder or recurrent mutations.

In controls, two *BRCA1* variants were found: c.5266dup and c.5251C>T. The founder mutation c.5266dup is known to be the most frequent in patients with BC, while the pathogenic variant c.5251C>T was described as a recurrent BC mutation in the Russian population. Both healthy carriers had a family history of BC and OC with affected first-degree relatives.

Forty P/LP variants were found in the *BRCA2* gene in both cohorts. At least six mutations were repeated and found in two or three unrelated individuals; thus, 16/40 (40%) cases in the total sample carried recurrent mutations, and 24/40 (60%) were found only once. A full list of P/LP *BRCA2* variants is presented in Table S2, and recurrent mutations are listed in Table 3. A previously undescribed nonsense variant, c.6980T>A (p.Leu2327Ter), was found in a 35-year-old patient with a family history of BC. Among 30 different types of P/LP variants in the *BRCA2* gene, 93% (28/30) were truncating mutations and only 7% (2/30) were missense (Tables S2 and S3).

In healthy controls, two pathogenic nonsense variants were found: c.2830A>T (p.Lys944Ter), which was also found in BC patients, and c.5286T>G (p.Tyr1762Ter). The carrier of c.2830A>T had a mother with colorectal and gastric cancer.

**Figure 4:**
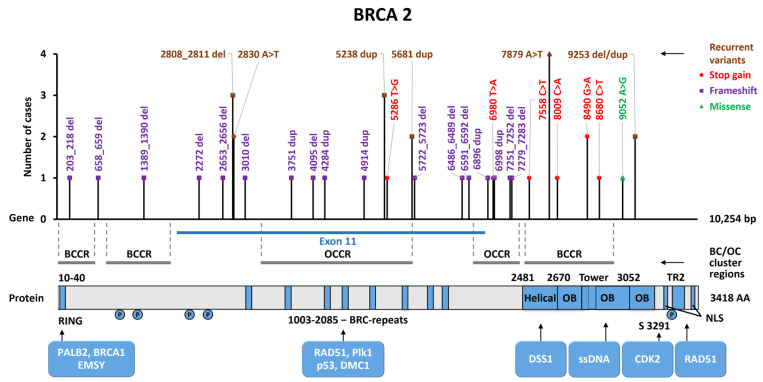
Location of P/LP variants found in the study along the *BRCA2* gene. Domain structure of the BRCA2 gene: RING—Really Interesting New Gene, BRC repeats—region consisting of approximately 1000 amino acids that bind to the protein Bacterial RecA homolog DNA recombinase (RAD51), Helical—Helical domain that comprises 190 amino acids, OB—Oligonucleotide/oligosaccharide-binding fold, Tower—Tower domain, TR2—C-terminal RAD51 binding site, NLS—Nuclear localization signal, P—Phosphorylation site. PALB2, BRCA1, EMSY, RAD51, PIk1, p53, DMC1, DSS1, CDK2 proteins, and ssDNA (single-stranded DNA) bind to BRCA2 at specific sites.

In Figure 4, all P/LP variants in the *BRCA2* gene from both cohorts are shown regarding the domain structure of the BRCA2 protein. The mutations are evenly distributed along the gene length, and 20/40 (50%) mutations are localized in the extended exon 11. No preferential localization of mutations in BC cluster regions was identified.

### 2.5. Spectrum and Frequency of P/LP Variants in Non-BRCA1/2 Genes

The *ATM* gene showed a statistically significant association (*p* < 0.05) with BC in our study (Table 2, Figure 2). The nonsense mutation c.5932G>T (p.Gln286Ter) was the most frequent in our cohort of BC patients (n = 5) (Table 4). The same mutation was found in one control case (a 49-year-old female with first-degree relatives with BC). Among eight types of P/LP variants, 4/8 (50%) were truncating mutations, 2/8 (25%) were splice site mutations, 1/8 (12.5%) was a missense mutation, and 1/8 (12.5%) was an in-frame insertion (Table S2). Three variants were not previously described in the *ATM* gene: the in-frame insertion c.3576_3576+7del and two splice variants, c.185+1G>T and c.4777-2A>C.

*CHEK2* was the gene that carried the highest number of pathogenic variants after *BRCA1* and *BRCA2* in BC patients (21/860 or 2.4%). Among 21 P/LP variants in the *CHEK2* gene found in BC patients, three main recurrent mutations were present: c.1100delC (10/21 or 48%), c.444+1G>A (6/21 or 29%), and c.433C>T (4/21 or 19%). The c.1100delC and c.444+1G>A variants were also revealed in controls, while c.433C>T was present only in patients. We observed no evidence of an association between P/LP variants in the *CHEK2* gene and BC risk (OR = 2.6 [95% CI: 1.0–6.9]). In addition, no statistical difference was found between the BC cohort and controls for c.1100delC (OR = 2.0 [95% CI: 0.5–7.40], *p* > 0.05) and c.444+1G>A (OR = 3.6 [95% CI: 0.4–30.4], *p* > 0.05).

All P/LP variants in the *PALB2* gene were truncating variants and occurred more frequently in the group of patients with BC compared to controls, with an OR =3.0 [95% CI: 0.4–26.1]; however, the difference was not statistically significant (*p* > 0.05). No repeated *PALB2* mutations were found in our sample. The variants *PALB2* c.509_510del, c.1291_1292del, and c.2936del were found earlier in families with BC or OC, while c.165del and c.2815_2822del were not described previously. In controls, the c.1240C>T mutation was identified in a 47-year-old carrier with a family history of BC (sister and grandmother were affected).

Other genes frequently discussed in the literature that are considered BC-associated genes are the *NBN*, *BLM,* and *MUTYH* genes. The P/LP variants in the *NBN* gene were presented only by a deletion of five nucleotides, c.657_661del, which was found in BC patients and in controls (OR = 1.2 [95% CI: 0.3–4.8], *p* > 0.05). The recurrent variant *FANCC* c.455dup (n = 3) was found only in BC patients, as well as the nonsense variant *NBEAL1* c.3463G>T (n = 5) (Table 4). For the *BLM*, *MUTYH*, and *FANCM* genes, the proportion of mutation carriers was very similar between patients and controls, while the pathogenic variants in the *SLX4* gene were slightly more prevalent in patients, although the difference was not significant (Table 2, Figure 2).

The comparative analysis of samples from breast cancer patients and healthy donors revealed a number of genes with P/LP variants only in the group of breast cancer patients. First, the *BARD1*, *RAD50*, *TP53*, and *XRCC2* genes, as well as the *RAD54B*, *ERCC2*, and *FANCI* genes, should be noted (Table S2). All mutations in the *BARD1* gene were presented by truncating variants; among them, a recurrent variant, c.1690C>T (p.Gln564Ter), was found. In the *RAD50* gene, four truncating variants and one affecting splice site were found (Table S2). Three patients had mutations in the *TP53* gene: two of them had early-onset BC (age at diagnosis: 29 and 30 years). In one case, bilateral BC developed in a 63-year-old woman. No *TP53* P/LP variants were found in controls (OR = 4.3 [95% CI: 0.2–82.4], *p* > 0.05).

For some genes, single P/LP variants were detected either only in the group of patients with breast cancer (*APC*, *MDM1*, *MRE11*, *POLD1*, *NF1*, *RAD51C*, *RECQL4*, and *WRN*) or only in the control group (*MCPH1*, *MSH3*, and *XPC*).

Eight patients (4%) from 190 positive individuals carried P/LP variants in two different genes (Table 5). It should be noted that in five out of eight cases, these were combinations of pathogenic variants in *BRCA1/2* genes with P/LP variants in other genes. The *NBN* gene was most often involved in such combinations; in four out of six cases of carrying the *NBN* mutation c.657_661del, a P/LP variant in another gene was also detected.

### 2.6. Correlations Between Genotype and Clinical Characteristics in BC Patients

Breast cancer patients with P/LP variants in *BRCA1*/*2* had, approximately, the same age at diagnosis as patients with P/LP variants in other genes, with average ages at diagnosis of 44.9 years (range from 24 to 77, ±11.6) and 46.7 years (range from 18 to 79, ±6.4).

**Figure 5:**
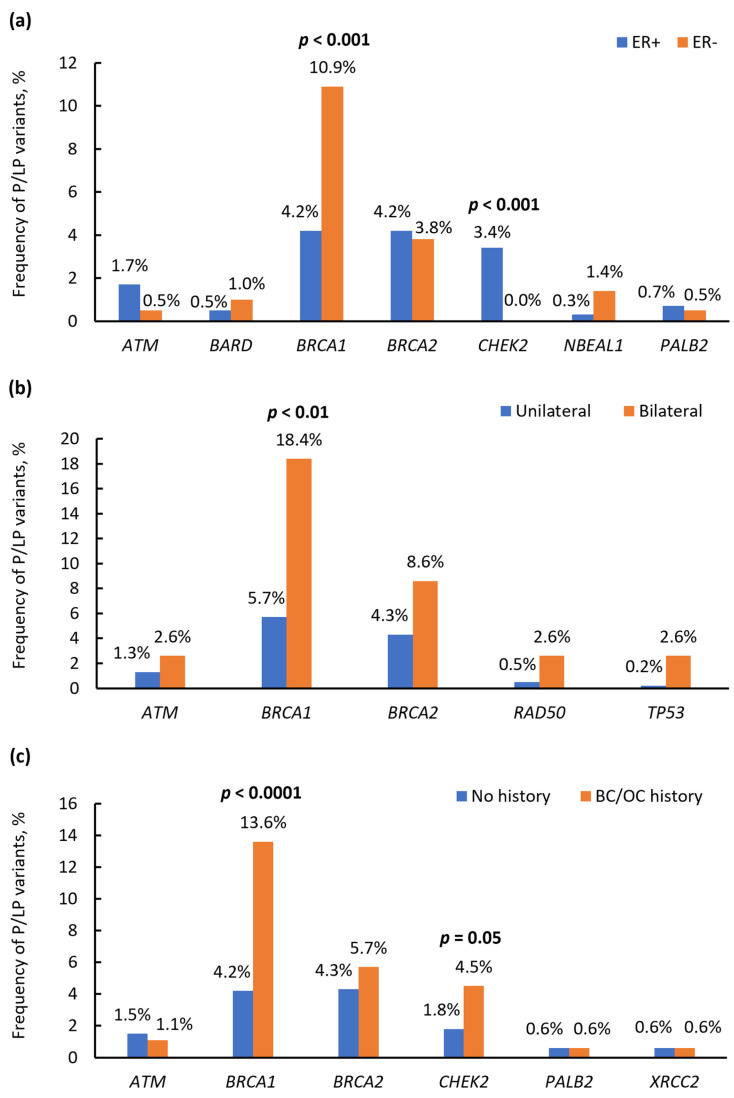
Association between P/LP variants in different genes and clinical features of patients with breast cancer: (**a**) estrogen receptor status, (**b**) bilaterality of breast cancer, (**c**) family history of breast or ovarian cancer.

Pathogenic/likely pathogenic variants in the *BRCA1* gene were found more often in patients with ER-negative BC compared to ER-positive BC (10.9% vs. 4.2%, *p* < 0.001), while P/LP variants in *CHEK2* were exclusively identified in BC patients with ER-positive breast cancer (3.4% vs. 0%, *p* < 0.001) (Table S4, Figure 5a). In addition, P/LP variants in the *BARD1* and *NBEAL1* genes were often found in patients with ER-negative BC, while variants in the *ATM*, *BRCA2*, and *PALB2* genes prevailed in ER-positive BC carriers, although the differences were not statistically significant (Table S4, Figure 5a).

In total, 38 patients (4.4%) had bilateral synchronous or metachronous breast cancer. Among patients with bilateral BC, carriers of P/LP variants were found more often than in patients with unilateral BC (16/38 or 42% vs. 182/822 or 22%, OR = 2.7 [95% CI: 1.4–5.3], *p* = 0.004). The most affected genes were *BRCA1* (8/16 or 50%), *BRCA2* (2/16 or 12.5%), and P/LP variants in the *ATM*, *TP53*, *BUB1*, *RAD50*, and *CTNNA1* genes. The pathogenic variants in the *BRCA1* gene were strongly associated with bilaterality of breast cancer with an OR = 4.4 [95% CI: 1.9–10.1], *p* = 0.002) (Table S5, Figure 5b).

We investigated the association between the presence of P/LP variants and a family history of BC or OC. Carriers with P/LP variants in the *BRCA1* gene were much more likely to have a family history of BC or OC than carriers of P/LP variants in other genes (OR = 3.57 [95% CI: 2.02–6.34], *p* < 0.0001). In addition, the association between the presence of P/LP variants in *CHEK2* and a family history of BC or OC was revealed (OR = 2.58 [95% CI: 1.04–6.41], *p* = 0.05) (Figure 5c). No associations were found between carrying P/LP variants in any of the genes studied and a family history of cancer other than BC or OC (Table S6).

Among BC patients, 34 females had primary multiple malignant tumors (PMMTs), including breast cancer. The most frequent additional tumor was gynecological cancer (ovarian cancer, endometrial cancer, and uterine cancer), which developed in 8/34 (24%) patients, thyroid cancer in 7/34 (21%) patients, and colorectal cancer in 5/34 (18%) patients. In addition, renal cancer, pancreatic cancer, Hodgkin lymphoma, lung carcinoma, melanoma, basal cell cancer, acute lymphoblastic leukemia, and non-Hodgkin lymphoma were diagnosed (Table S7). Seven patients with PMMTs had three primary tumors. Among them, three patients had bilateral BC (Table S7). 

Among 34 patients with PMMTs, P/LP variants were found in 9/34 (26%) compared to 189/826 (23%) in patients with only BC (unilateral or bilateral). The affected genes were *ATM*, *BRCA2*, *CHEK2*, *MUTYH*, *FANCC*, and *RAD52* (Table 6)

The P/LP variants in the *ATM* gene were found more frequently in patients with PMMTs compared to patients with BC alone (8.8% vs. 1.0%, OR = 8.8 [95% CI: 2.3–34.1], *p* = 0.01) (Table S8). The spectrum of malignancies included breast cancer, colorectal cancer, and thyroid cancer. Two patients were carriers of the known mutation *ATM* c.5932G>T. Two patients had non-recurrent *BRCA2* mutations: one carrier had colorectal cancer and synchronous BC and OC, and the other had early-onset synchronous BC and lung carcinoma. The pathogenic variant c.455dup in the *FANCC* gene was found in a patient with Hodgkin lymphoma and breast cancer (Table 6).

## 3. Discussion

The identification of clinically significant genetic variants associated with hereditary breast and/or ovarian cancer is essential in an era when medicine is focused on each patient to ensure the most appropriate treatment, prognosis, and follow-up. It is also important for medical counseling for close relatives, as the identification of the pathogenic allele as the cause of the disease makes it possible to assess the risk of the disease for family members and to propose a personalized approach to prevention [32,33]. Many studies have demonstrated that each local population is characterized by the prevalence of certain genetic variants [28,29,30,31,32,34,35,36]. Therefore, the study of specific genetic loads for inhabitants of different geographical areas and different ethnicities allows the most appropriate panel of genetic markers to be chosen, leading to a more accurate interpretation of the results of genetic tests.

In our study, the recruitment of breast cancer patients was based on the patient’s referral to the Personalized Medicine Center for genetic testing for individualization of treatment and medical and genetic counseling. In this regard, the majority of the sample consisted of patients aged 50 years or younger (562/860 or 65.3%), since it is in younger patients that breast cancer is associated with hereditary mutations and genetic testing is most relevant [33,37,38]. In addition, a notable proportion of patients (8%) had more than one tumor (bilateral breast cancer or malignancies of other localizations), and 21% of patients had a family history of BC and/or OC. To better evaluate the contribution of mutations in various genes to the risk of BC development, we used a group of healthy women matched for age and family history.

**Figure 6:**
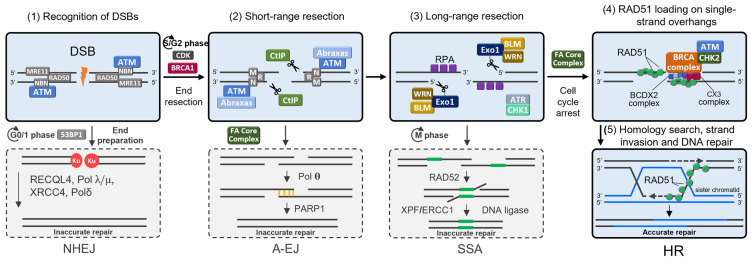
Schematic representation of protein interactions involved in DNA DSB repair pathways. DSBs in DNA can be repaired by non-homologous end-joining (NHEJ), alternative end-joining (A-EJ), single-strand annealing (SSA), or HR. Multigene panel included 48/78 genes coding the key proteins involved in these DNA damage repair pathways. (1) Repair begins with recognition of DSB sites by the MRN complexes (MRE11-RAD50-NBN), which recruit Ataxia telangiectasia mutated (ATM) kinase. Depending on cell cycle and cyclin-dependent kinase (CDK) activity, ATM promotes different repair pathways. In the G0 and G1 phases, CDK activity is low and ATM phosphorylates 53BP1, favoring EJ pathways with almost no processing of DSB ends, which results in short nucleotide insertions and deletions at the sites of repair. In the S and G2 phases, CDK activity increases and ATM phosphorylates BRCA1, allowing DSB end resection. (2) C-terminal binding protein-interacting protein (CtIP) and the MRN complex lyse DNA strands from 5′ to 3′, generating stretches of single-stranded DNA (ssDNA) at DNA ends for A-EJ, SSA, and HR. Ablation of the BRCA1–Abraxas interaction with these complexes promotes extensive resection. (3) Bloom syndrome RecQ-like helicase (BLM), WRN RecQ-like helicase (WRN), and exonuclease 1 (EXO1) account for additional resection, forming overhanging ssDNA ends that can invade the homologous template. Replication protein A (RPA) binds to ssDNA, activating the ATM RAD3-related (ATR) kinase, ATR phosphorylates checkpoint kinase 1 (CHK1), which arrests the cell cycle, allowing time for DSB repair. In the M phase, both HR and EJ are blocked and DSBs arising during mitosis are repaired by SSA, which results in large-scale chromosomal rearrangements. (4) ATM-phosphorylated checkpoint kinase 2 (CHK2) recruits the BRCA complex, consisting of BRCA1/2, PALB2, BRIP1, BARD1, and SLX4. The BRCA complex regulates the interaction of RAD51 paralog complexes BCDX2 (RAD51B–RAD51C–RAD51D-XRCC2) and CX3 (RAD51C–XRCC3) with RAD51 recombinase. RAD51 is loaded on ssDNA to form the RAD51–ssDNA nucleofilament. (5) RAD51 catalyzes the homology search and synthesizes DNA using sister chromatids as a template, which ensures a low error likelihood. The Fanconi anemia (FA) Core Complex (FANCA, B, C, E, F, G, L, M, and T) activates FANCD2 and FANCI by mono-ubiquitinating the proteins as a response to DNA damage. The activated FANCD2–I heterodimeric proteins are subsequently transported to the sites of DNA repair, which contain FA downstream proteins of the BRCA complex. Polδ, coded by the *POLD1* gene, is involved in replication-coupled DNA events associated with repair, including NHEJ and break-induced recombination (blocks highlighted in light blue illustrate the main steps of HR, key participating proteins are marked in other colors).

Another important consideration was the number of genes included in our panel for target sequencing. Several laboratories have released commercial multigene panels ranging from six to >100 genes [39]. In most studies, multigene panels include about 20–40 genes associated with hereditary cancer syndromes [8,9,13,14,16,22]. Recently, 657 patients from Russia with clinical signs of hereditary cancer syndromes were tested using a multigene hereditary cancer panel of 44 genes [10]. In our multigenic panel, we included genes involved in DNA repair processes, above all those involved in the repair of DNA double-strand breaks (DSBs) by homologous recombination (HR), since it is known that disruption of these processes leads to the development of BC and/or OC [40,41]. In total, we investigated the coding regions of 78 genes, resulting in 190 out of 860 BC patients (22%) being carriers of 198 P/LP variants in 30 genes, of which eight patients had two P/LP variants each in different genes. Among 520 healthy controls, only 6.2% of individuals carried P/LP variants in 17 genes. Thus, for the entire pool of P/LP variants, there was a prominent and statistically significant difference between BC patients and healthy individuals (23% vs. 6.2%, OR = 4.5, *p* < 0.0001), and it was even larger when we considered only truncating variants (18.1% vs. 4.4%, OR = 4.8, *p* < 0.0001). In BC patients, 21/30 or 70% of damaged genes were participants in the repair processes of DSBs in DNA, mainly in HR (Figure 6) [40,42,43].

Other affected genes were involved in nucleotide excision repair (*ERCC2*), base excision repair (*MUTYH*), or cellular signaling pathways (*APC*, *CTNNA1*, *TP53*, *MDM1*, *NF1*, *BUB1*, and *NBEAL1*). Next, we examine the possible contribution of individual genes and P/LP variants to BC risk in comparison with other studies.

### 3.1. BRCA1 and BRCA2 Genes

BRCA1 is a multifunctional protein and a key player in both checkpoint activation and DNA repair, while BRCA2 is an important mediator of homologous recombination [38,39,40]. These are essential activities to prevent tumor development, which determine their leading role in predispositions to breast or ovarian cancer. In our study, frequencies of *BRCA1* and *BRCA2* mutations in BC patients were 6.3% and 4.4%, and ORs were 16.3 and 12.0, respectively. These estimates are higher than estimates in unselected population samples of women with BC [21,23,44], which may be explained by the prevalence of patients with early-onset cancer (<50 years).

It was shown in many studies that Slavic populations are characterized by a high incidence of *BRCA1* founder mutations, while *BRCA2* mutant recurrent alleles contribute only to a minor fraction of BC and OC incidence [24,25,26,27,28,45]. In our study, eight known founder mutations in the *BRCA1* gene contributed to 71% of all cases. In addition, three recurrent mutations were found: c.4675G>C, c.4689C>G, and c.1303_1309del, which accounted for an additional 11%. The variant c.4689C>G was earlier described in the Russian population, along with c.5251C>T [28], thus allowing us to consider them as candidate founder mutations in the Slavic population of Russia. The variant c.4675G>C was described in a Polish study of OC cases. The variant *BRCA1* c.1303_1309del was previously found in an Israeli patient [46]. The *BRCA1* variants, which were unique in our study, comprised 11% of all cases and were described in different ethnic groups worldwide. An LP variant, c.4507del (p.Ser1503HisfsTer2), was not previously described in other studies on BC patients. It is a frameshift mutation resulting in truncation of the BRCA1 protein chain and loss of both BRCT domains; the carrier had triple-negative BC at the age of 37 years and there were two BC cases in the family (one was her sister). The P/LP variants in *BRCA1* were strongly associated with ER-negative breast cancer (*p* < 0.001), bilaterality (*p* < 0.01), and a family history of BC/OC (*p* < 0.0001), which is consistent with other studies [5,13,28,37]. The majority of *BRCA1* mutations (83%) were located in so-called BC cluster regions (BCCRs), and about 17% were found in OC cluster regions (OCCRs) [38].

In the *BRCA2* gene, six recurrent mutations were revealed, contributing to 40% of cases. Among them, variant c.7879A>T (p.I2627F) was the most frequent (10% of all identified *BRCA2* variants). Previously, this variant was described in BC or OC patients from Russia [28,47,48], and also in populations from Slovenia [49] and Macedonia [50]. Thus, mutation c.7879A>T can be considered a founder mutation for the Slavic population in Russia. Another mutation, c.2808_2811del, was also found in 1399 ovarian cancer patients recruited from 72 Russian regions [47]. However, in general, it can be said that almost every study identifies its own recurrent mutations. The P/LP *BRCA2* variants were found more frequently in patients with bilateral BC and in patients with a family history of BC/OC, but the associations were not significant. Among 34 individuals with PMMTs, pathogenic variants in the *BRCA1* gene occurred in two cases (6%); the other tumors were colorectal and ovarian cancer in one case, and lung cancer in another. Mutations were distributed evenly along the length of the *BRCA2* gene and there was no preferred localization in the BCCRs.

### 3.2. ATM, CHEK2, PALB2, and BARD1 Genes

The ataxia–telangiectasia mutated (ATM) gene encodes a serine/threonine kinase involved in DNA double-strand break repair pathways [40,41]. Bi-allelic deleterious variants in the *ATM* gene cause a rare autosomal recessive neurodegenerative disease, ataxia–telangiectasia, which occurs in approximately 1 per 880,000 live births [51]. Heterozygosity for loss-of-function variants in *ATM* has been shown to be associated with an increased risk of development BC, as well as a significant risk of prostate, pancreatic, and ovarian cancers [51,52].

Usually, the *ATM* gene is considered as a moderate risk gene for BC predisposition. In large cohort studies, the OR for the *ATM* gene varies from 2.1 [21] to 3.38 [41] in BC patients. One meta-analysis reported that truncating and missense variants confer an estimated BC relative risk (RR) of 2.8%, and an absolute BC risk by 80 years of age of 27% [53,54,55]. In our study, *ATM* P/LP variants are significantly associated with BC risk, with an OR = 7.3 (*p* = 0.037). This is more consistent with another meta-analysis of 19 studies by Marabelli M. et al. [56], which reported that the female carriers of *ATM* variants have a breast cancer relative risk of 6.02 by the age of 50 years old and 32.83 by the age of 80 years old. The most frequent variant, c.5932G>T, was found in 5/12 (42%) BC patients and in one control with a family history of BC. This variant was described as a founder mutation, which predisposes to BC in populations in Belarus, Russia, Ukraine, and Poland [57,58]. P/LP variants in *ATM* were more common, but not significantly in ER-positive BC, which is consistent with other studies [21,22]. We found a significant association between the presence of truncating variants c.5932G>T and c.4148C>A in the *ATM* gene and the development of other primary tumors besides BC (colorectal or thyroid cancer), with an OR= 8.8 (*p* = 0.01).

Clinically significant variants in the *CHEK2* gene were the most frequently identified after P/LP variants in the *BRCA1* or *BRCA2* genes in our study, which is consistent with many other studies [3,6,21,23]. In our study, the association between *CHEK2* P/LP variants and risk of BC, with an OR= 2.6, did not reach statistical significance, but the risk was confirmed in other studies with larger cohorts. The prevalence of *CHEK2* c.1100delC in some populations makes it possible to consider the risk associated with this variant individually. In a study of 11,416 affected women, estimates had an OR = 4.00 for *CHEK2* c.1100delC and an OR = 1.42 for all other *CHEK2* P/LP variants [59]. Another population-based study estimated an OR of 2.66 for *CHEK2* c.1100delC and an OR of 2.13 for all other *CHEK2* P/LP variants [22]. In our study, c.1100delC was also present in healthy controls (n = 5); thus, for c.1100delC alone, the OR was 2.0. For the other variant c.444+1G>A, the OR was 3.6, which was not significant. *CHEK2* P/LP variants were represented mainly by three mutations: c.1100delC, c.444+1G>A, and c.433C>T. All of them were previously described in populations of Slavic origin as founder mutations [60,61]. Earlier, it was found that CHEK2*1100delC heterozygosity in women with BC was associated with early death, breast cancer-specific death, and increased risk of recurring BC [62,63]. In our study, *CHEK2* mutations were associated with ER-positive BC (*p* < 0.001) and a family history of BC/OC (*p* = 0.05), but no associations were found with the presence of bilateral BC.

The *BARD1* gene is the main partner of the *BRCA1* gene, while the *PALB2* gene is the main partner of the *BRCA2* gene. Many studies have estimated the contribution of *BARD1* mutations in breast cancer susceptibility [64,65]. Previously, the association of truncating variants in the *BARD1* gene with risk of BC was reported with an OR = 1.37 (*p* > 0.05) [21] and an OR = 2.09 (*p*< 0.05) [22]. In our study, all P/LP *BARD1* variants were truncating and not found in the control group, but the association with the risk of BC was not significant, with an OR= 6.7. The repeated *BARD1* variant c.1690C>T (p.Gln564Ter) has been previously described as a recurrent mutation in other ethnic groups, including Slavic populations [66].

We found that P/LP variants in the *PALB2* gene are associated, although not significantly, with the risk of breast cancer (OR = 3.0). All *PALB2* mutations were truncating variants, and the variant c.509_510del was previously described as a founder mutation in Slavic populations [67,68]. In controls, another Slavic recurrent c.1240C>T mutation (p.R414*) was identified in a 47-year-old woman with a family history of BC and an affected first-degree relative. In international studies with large cohorts, pathogenic variants in *PALB2* were significantly associated with a moderate risk of BC, with ORs from 3.83 [21] to 5.02 [22].

### 3.3. NBN, RAD50, and MRE11

The MRN complex (MRE11–RAD50–NBS1) is responsible for initiating DNA DSB repair and activating several downstream proteins (Figure 6). In our study, the well-known Slavic founder mutation *NBN* c.657_661del5 was present in BC patients and in healthy controls (0.7% vs. 0.6%, OR = 1.2, *p* > 0.05). Many studies were published to evaluate the association between the *NBN* c.657_661del5 variant and BC risk, but the results remained inconsistent. Earlier, a significant association with breast cancer was reported [69], but in later studies, no increased risk of breast cancer was observed among participants with any pathogenic variant in *NBN* (OR = 1.05) or with the *NBN* pathogenic variant c.657_661del5 (OR = 0.93) [22]. Furthermore, in our study, three BC patients with *NBN* c.657_661del5 also carried *BRCA1* or *BRCA2* pathogenic variants, which had to contribute more significantly to the development of the disease.

We found P/LP variants in the *RAD50* gene only in BC patients. The OR was 6.7, but not significant. In other studies, no increased risk for BC was reported, with an OR = 1.08 [22]. On the other hand, in vitro experiments showed cell cycle arrest in the G2/M phase, leading to death by apoptosis in RAD50-deficient cells treated with PARP inhibitors alone or in combination with carboplatin [70]. In addition, *RAD50* germline mutations were found to be associated with poor survival in *BRCA1/2*-negative BC patients [71].

We found only one LP variant in the *MRE11* gene. In large-cohort studies, no association was revealed between *MRE11* mutations and BC risk (OR = 0.88) [22].

### 3.4. Fanconi Anemia Genes (FANCC, FANCI, FANCM, and SLX4)

In our study, moderately increased BC risk was defined for mutations in the Fanconi anemia genes *FANCC* (OR = 2.4) and *FANCI* (OR = 4.3), but not for *FANCM* (OR = 0.6), although the difference was not significant. In large cohorts, no increased risk for Fanconi anemia genes was found [22,72]. On the other hand, we should note the pathogenic variant c.455dup in the *FANCC* gene, which was revealed only in BC patients in our study. Previously, the variant was described mainly in families with Fanconi anemia [73].

The *SLX4* gene encodes a DNA repair protein that regulates three structure-specific endonucleases and is necessary for resistance to DNA crosslinking agents, topoisomerase I, and poly (ADP-ribose) polymerase (PARP) inhibitors. Recent studies have reported mutations in the *SLX4* gene in a new subtype of Fanconi anemia (FA), FA-P. Loss-of-function mutations in *SLX4* may contribute to the development of breast cancer in very rare cases [74]. We found three truncating variants in BC patients, and one truncating variant in controls, so the results were inconsistent due to the rarity of the variants (OR = 1.8, *p* > 0.05).

### 3.5. RAD51C and XRCC2 Genes

The RAD51 recombinase catalyzes DNA strand exchange and ultimately DNA repair during HR by forming nucleoprotein filaments on single-stranded DNA at sites of double-stranded breaks. Five RAD51 paralogs, RAD51B, RAD51C, RAD51D, XRCC2, and XRCC3, are also involved in this process. RAD51C forms two distinct complexes: BCDX2 (RAD51B–RAD51C–RAD51D–XRCC2) and CX3 (RAD51C–XRCC3), which act at two different stages of homologous recombinational DNA repair. The BCDX2 complex is responsible for the recruitment of RAD51 at the sites of damage and ensures the search for a homologous DNA strand, as well as the invasion of the strand for subsequent completion [42]. Thus, deleterious mutations in genes encoding these proteins are considered to predispose to BC and OC [75]. We found the variant *RAD51C* c.224_225dup, previously described in BC and OC families [76], in a 51-year-old patient with no family history of BC.

Another paralog of RAD51 is XRCC2, encoded by the *XRCC2* gene, which has a disputable relationship to breast cancer risk. Different truncating variants in *XRCC2* showed no evidence for association with BC risk in several studies [77]. The variant *XRCC2* c.96delT (p.F32fs) was described as a protein-truncating founder variant in Poland and no evidence was found that this mutation predisposed to BC or other cancers [78]. In our study, c.96delT was detected in six unrelated BC patients (0.7%), with an OR = 7.9 (*p* > 0.05), and none in the control group. Moreover, it was present in a familial case of BC, where both the daughter and mother carrying this variant had breast cancer.

### 3.6. NBEAL1 Gene

The *NBEAL1* gene encodes the BEACH (Beige and Chediak–Higashi) domain protein Neurobeachin-like 1, which is a Golgi-associated protein required for the regulation of cholesterol metabolism. Exome sequencing in HBOC families of Greek and Canadian origin uncovered rare loss-of-function variants in the *NBEAL1* gene [79]. The variant c.3463G>T (p.E1155*), with MAF = 0.06%, was mentioned as a potential risk marker for breast cancer susceptibility. The same variant was revealed in our study exclusively in BC patients (0.6%, OR = 3.0, *p* > 0.05). The variant c.3463G>T was classified as likely pathogenic according to ACMG and was associated with ER-negative BC (not significant).

### 3.7. MUTYH Gene

The *MUTYH* gene encodes a base excision repair DNA glycosylase that helps protect cells against the mutagenic effects of guanine oxidation [80]. A series of clinical observations have shown that biallelic and heterozygous germline pathogenic variants in *MUTYH* are associated with the development of familial adenomatous polyposis [81]. In the last years, several studies also investigated the impact of germline monoallelic *MUTYH* pathogenic variants in genetic susceptibility to the development of BC, OC, or prostate cancer, showing increased risk rates associated with heterozygosity [79]. The association between monoallelic *MUTYH* pathogenic variants and the risk of BC and OC remains controversial [82,83]. In our study, the proportion of *MUTYH* carriers was the same in BC patients (0.8%) and controls (0.8%), with an OR = 1.06. The most frequent mutations were c.1103G>A (p.G368D) (MAF = 0.0041) and c.650G>A (p.R217H) (MAF = 0.01).

### 3.8. ERCC2 and Other Non-BRCA1/2 Genes

*ERCC2* is a DNA repair gene involved in the nucleotide excision repair pathway, which encodes the DNA helicase XPD (xeroderma pigmentosum group D). Several polymorphisms in the *ERCC2* gene were widely studied for BC susceptibility, and a meta-analysis showed an increased BC risk for the *ERCC2* variant Lys751Gln [84]. We found three different P/LP variants in the *ERCC2* gene only in BC patients, and the variants were previously defined in patients with xeroderma pigmentosum or trichothiodystrophy in a homozygote state. A variant, c.1703_1704del (p.Phe568Tyrfs), was also described as a recurrent heterozygous mutation in BC patients [85]. However, the contribution of *ERCC2* genetic variants to BC susceptibility remains inconsistent.

Mutations in the *TP53* gene were found in 0.3% of BC patients, most probably associated with Li–Fraumeni syndrome. Among BC patients, very rare P/LP variants were found in the *APC*, *CTNNA1*, *MDM1*, *POLD1*, *NF1*, *RAD54B*, *RECQL4*, and *WRN* genes. In addition, in controls, single pathogenic variants in the *MCPH1*, *MSH3*, and *XPC* genes were revealed. Because of the rarity of these events, we are unlikely to draw definite conclusions about the contribution of these genes to breast cancer susceptibility. On the other hand, individual consultations with patients and segregation analysis may allow new susceptible loci and pathogenic variants to be revealed, as well as comparisons with data from the literature.

Mutations in some genes were described more often in other hereditary cancers. We identified a nonsense mutation in the *CTNNA1* gene in a 55-year-old patient with bilateral BC. Loss-of-function variants of the *CTNNA1* gene have been described predominantly in patients with gastric cancer, and more rarely in patients with breast cancer [86]. The LP variant affecting the splice site in the *POLD1* gene was found in a 41-year-old patient with a mother diagnosed with BC. Germline mutations in the *POLE* and *POLD1* genes, coding for DNA polymerases ε and δ, were described to be associated mainly with an increased risk of polyposis and colorectal cancer. In addition, *POLD1* mutations were found to predispose to endometrial and breast tumors [87]. Furthermore, POLE/POLD1-mutated tumors show a high tumor mutational burden, and the identification of these genetic alterations could be used to select patients who are suitable for immunotherapy treatment. *APC* is another gene known to be associated with hereditary polyposis and colorectal cancer. In our study, the variant c.3920T>A (p.Ile1307Lys) in the *APC* gene was identified in a 26-year-old BC patient with no family history of BC. This variant has been reported as a candidate low-penetrance BC risk factor or genetic modifier in *BRCA1/2* cases [88], and it has also been found in male patients with BC [89]. Further observation of the patient and her family may elucidate the role of the *APC* variant in the personal history of cancer. The cases described also raise the question of whether the deleterious mutation found in these genes increases the risk of developing a cancer other than breast cancer.

Next-generation sequencing allows simultaneous testing of multiple genes that have different estimated risks for breast or other cancers. For patients with breast cancer, the detection of mutations in homologous recombination genes also provides a rationale for the use of PARP inhibitors. With the increasing scale of genetic testing, commercial multigene panels with an expanded number of genes to be evaluated are becoming more common, raising questions about whom to test and which genes should be included. According to the results of our study, as well as other studies, the majority of deleterious variants identified are in the most recognized genes, such as *BRCA1*, *BRCA2*, *ATM*, *CHEK2*, *PALB2,* and *TP53*. For other genes, results cannot always be interpreted unambiguously, especially in the case of rare variants in genes poorly studied in terms of breast cancer risk. Nevertheless, genetic screening with a wider range of genes may be of interest because it may reveal new links between genes and disease. It is also possible to identify characteristic features of breast cancer cells carrying a mutation in this gene, which will make it possible to search for new approaches to therapy.

The study of local populations allows a better understanding of their genetic structure and the development of more adequate approaches to genetic testing. In the large population we studied, a sufficiently high percentage of new recurrent mutations in both *BRCA1/2* and non-*BRCA1/2* genes can be observed that can also be included in routine screening by PCR methods. This approach could involve a much larger section of the population to form groups of individuals at increased risk of breast cancer and further expand mammographic screening for early cancer detection [90].

Our study has several limitations. First, we had no tumor material from the patients carrying pathogenic variants in order to investigate corresponding alterations, such as homologous recombination deficiency or activation of signaling pathways, in breast cancer cells. Functional assays can provide important additional information for variant classification but are currently less well-developed for breast cancer predisposition genes other than *BRCA1* and *BRCA2*. A second limitation is that the size of our sample was not large enough to reach statistical significance in the case of rare candidate genes and rare variants. The third limitation is related to the peculiarities of the formation of our patient cohort, as some younger patients were selected for testing. Thus, our data do not fully reflect the risks of breast cancer development in the general population.

## 4. Materials and Methods

### 4.1. Patients

The study cohort included 860 women with breast cancer and 520 healthy women, who are residents of the regions of the Central Federal District of Russia. Participation in this study included molecular genetic testing, for which all participants provided detailed information about their personal and family history of cancer. For testing, each participant provided two blood samples, each collected in a 2 mL EDTA tube. Genomic DNA was isolated from leukocytes using the QIAamp DNA Blood Mini Kit from Qiagen (Qiagen, Hilden, Germany) according to the manufacturer’s instructions.

### 4.2. Genetic Testing

To establish the multigene panel, we selected 78 genes known to be involved in DNA repair processes, primarily repair by homologous recombination. The complete list is presented in Table S1. The panel was designed using the HyperDesign online service provided by Roche (Roche, Basel, Switzerland), which included exons of selected genes and adjacent intronic regions containing splicing sites. The probe selection was performed using the reference genome hg19/GRCh37, Homo sapiens. The panel included 1750 gene regions with a total sequence length of 0.4 Mb. The percentage of target bases was estimated to be 99.13%. Libraries were prepared using the KAPA HyperPrep Kit (Roche, Basel, Switzerland), and for targeted sequence selection, hybridization with a custom panel was performed following the manufacturer’s protocol KAPA HyperCap Workflow v3.0 (Roche, Basel, Switzerland). Sequencing was performed on the MiSeq platform from Illumina (Illumina, San Diego, CA, USA) using the MiSeq Reagent Kit v2 with 300 cycles, paired-end and with an average coverage of 150–200×.

### 4.3. Bioinformatics and Statistical Analysis

The quality of sequencing was estimated using the FastQC tool. Paired-end reads were aligned to the reference genome (hg37) using the BWA-MEM2 algorithm. Duplicate sequences were then identified and removed using the Picard MarkDuplicates program. Then, recalibration of the quality scores of the bases was performed and identification of genetic variants was performed using Genome Analysis Toolkit (GATK) tools: BQSR for recalibration of scores and HaplotypeCaller for variant calling. The uniformity of base coverage exceeded 98% for all samples. All samples with an average coverage ≥ 50× were included in the further analysis. Germline variants were recorded if they passed all HaplotypeCaller filters and the total number of reads covering them was ≥50. Variant annotation was performed using wANNOVAR (https://wannovar.wglab.org) or OpenCRAVAT (https://run.opencravat.org) software. Finally, all variants of interest were analyzed using an Integrative Genomic Viewer (IGV). Interpretation of all identified variants was performed using open-access databases: ClinVar, Varsome, and Franklin by Genoox, which utilize the American College of Medical Genetics and Genomics and the Association for Molecular Pathology (ACMG-AMP) interpretation standards and guidelines [91]. Variants were classified as benign (B), likely benign (LB), variant of uncertain significance (VUS), likely pathogenic (LP), or pathogenic (P). A variant was considered for further investigation if it was categorized as “pathogenic “or “likely pathogenic” in at least one database.

To verify the NGS data, direct sequencing was used for about 20% of the variants identified. The primers for the region of interest were selected using the Primer-BLAST designing tool [92]. The PCR mixture (final volume of 25 μL) consisted of 1× Hot Start Taq-DNA polymerase buffer; 2 mM MgCl2; 1 unit Hot Start Taq DNA polymerase (SibEnzyme, Nowosibirsk, Russia); 0.2 mM dNTPs; 0.04 μM of forward and reverse primers; and 20–30 ng DNA. The PCR cycling conditions were as follows: 3 min at 95 °C, followed by 35 cycles of 30 s at 95 °C, 30 s at 60 °C, 30 s at 72 °C, and then 72 °C for 2 min. Sanger sequencing was performed using a 3500 Series Genetic Analyzer (Applied Biosystems, Thermo Fisher Scientific, Waltham, MA, USA).

Statistical analysis was performed using the two-sided Fisher’s criterion.

## 5. Conclusions

The results of this study provided new information on the role of pathogenic variants in various genes involved in the processes of DNA repair in the development of breast cancer. We have described rare variants associated with breast cancer risk and identified new recurrent mutations that will improve and optimize the strategy of genetic testing for breast cancer susceptibility. According to our data, genetic testing of non-*BRCA1/2* genes may double the yield of clinically significant variants. In cases of very rare genetic variants, individual consultations with patients and segregation analysis of a family may allow new susceptible loci and pathogenic variants to be revealed.

## Figures and Tables

**Table 1:** Descriptive statistics for the subjects included in this study.

Clinical Characteristics	Patients with BC	Healthy Controls
Number of subjects	860	520
Cases, n (%)	860 (100%)	-
Cases diagnosed at age 50 years or younger, n (%)	562 (65.3%)	-
Controls, n (%)	-	520 (100%)
Average age in years at diagnosis (for patients) or at time of testing (for controls), median (min–max)	48 (18–83)	51 (24–87)
Female, n (%)	860 (100%)	520 (100%)
Estrogen receptor status of tumor (available), n (%)	800 (100%)	-
ER-positive breast cancer, n (%)	590 (74.0%)	-
ER-negative breast cancer, n (%)	210 (26.0%)	-
Molecular subtypes of breast cancer (available), n (%)	781 (100%)	-
Luminal A	193 (25.0%)	-
Luminal B (Her2-positive)	95 (12.0%)	-
Luminal B (Her2-negative)	279 (36.0%)	-
Her2-positive	47 (6.0%)	-
Triple-negative	167 (21.0%)	-
Personal history of other cancers including BC, n (%)	69/860 (8.0%)	-
Bilateral BC, n (%)	38/860 (4.4%)	-
Primary multiple tumors including BC, n (%)	34/860 (4.0%)	-
Family history of cancer (available), n (%)	843 (100%)	520 (100%)
Presence of relatives with cancer diagnosis, n (%)	360 (43.0%)	236 (45.0%)
No cases with any cancer in the family, n (%)	483 (57.0%)	278 (55.0%)
Relatives with BC or ovarian cancer, n (%)	178 (21.0%)	94 (18.0%)
First-degree relatives with BC or ovarian cancer, n (%)	100 (12.0%)	68 (13.0%)
First-degree relatives with other cancers, n (%)	151 (18.0%)	111 (21.0%)

**Table 2:** P/LP variant carriers identified by multigene panel testing, with the ORs and corresponding 95% confidence intervals (CIs) for associations with breast cancer (*—2 from 7 mutations in *XRCC2* were found in one family).

Gene	Patients (n = 860)		Controls (n = 520)		OR (95% CI)	*p*-Value
	Number of Carriers	%	Number of Carriers	%		
*ABRAXAS1*	1	0.1	1	0.2	0.6 (0.04–9.68)	1.00
*APC*	1	0.1	0	0.0	1.8 (0.1–44.7)	1.0
*ATM*	12	1.4	1	0.2	7.3 (0.9–56.7)	0.037
*BARD1*	5	0.6	0	0.0	6.7 (0.4–121.4)	0.16
*BLM*	2	0.2	3	0.6	0.4 (0.07–2.4)	0.37
*BRCA1*, including	54	6.3	2	0.4	16.3 (4.0–66.7)	<0.0001
5266dup	25	2.9	1	0.2	15.5 (2.1–115.1)	0.0001
*BRCA2*	38	4.4	2	0.4	12.0 (2.9–45.9)	<0.0001
*BUB1*	1	0.1	1	0.2	0.3 (0.03–3.3)	0.56
*CHEK2*	21	2.4	5	1.0	2.6 (1.0–6.9)	0.06
*CTNNA1*	1	0.1	0	0.0	1.8 (0.1–44.7)	1.0
*ERCC2*	3	0.3	0	0.0	4.3 (0.2–82.4)	0.23
*FANCC*	4	0.5	1	0.2	2.4 (0.3–21.8)	0.66
*FANCI*	3	0.3	0	0.0	4.3 (0.2–82.4)	0.23
*FANCM*	2	0.2	2	0.4	0.6 (0.1–4.3)	0.63
*MDM1*	1	0.1	0	0.0	1.8 (0.1–44.7)	1.0
*MRE11*	1	0.1	0	0.0	1.8 (0.1–44.7)	1.0
*MUTYH*	7	0.8	4	0.8	1.06 (0.3–3.6)	1.0
*NBEAL1*	5	0.6	1	0.2	3.0 (0.3–26.0)	0.4
*NBN*	6	0.7	3	0.6	1.2 (0.3–4.8)	1.00
*NF1*	1	0.1	0	0.0	1.8 (0.1–44.7)	1.0
*PALB2*	5	0.6	1	0.2	3.0 (0.4–26.1)	0.42
*POLD1*	1	0.1	0	0.0	1.8 (0.1–44.7)	1.0
*RAD50*	5	0.6	0	0.0	6.7 (0.4–121.4)	0.16
*RAD51C*	1	0.1	0	0.0	1.8 (0.1–44.7)	1.0
*RAD54B*	2	0.2	0	0.0	3.0 (0.2–63.4)	0.53
*RECQL4*	1	0.1	0	0.0	1.8 (0.1–44.7)	1.0
*SLX4*	3	0.3	1	0.2	1.8 (0.2–17.5)	1.00
*TP53*	3	0.3	0	0.0	4.3 (0.2–82.4)	0.23
*WRN*	1	0.1	0	0.0	1.8 (0.1–44.7)	1.0
*XRCC2*	7 (6) *	0.8 (0.7)	0	0.0	7.9 (0.4–140.9)	0.08

**Table 3:** Spectrum of founder mutations and recurrent P/LP variants in the *BRCA1* and *BRCA2* genes in BC patients and controls. Variants present in both patients and controls are marked by (*).

Gene	c.HGVS	Type	SNP ID	n (%)	ACMG
*BRCA1*	Founder mutations				
	c.5266dup (*)	frameshift	rs80357906	26 (45%)	P
	c.4035del	frameshift	rs80357711	3 (5.5%)	P
	c.181T>G	missense	rs28897672	3 (5.5%)	P
	c.1961del	frameshift	rs80357522	3 (5.5%)	P
	c.3700_3704del	frameshift	rs80357609	1 (1.8%)	P
	c.68_69del	frameshift	rs80357914	1 (1.8%)	P
	c.3847_3848del	frameshift	rs80359405	1 (1.8%)	P
	c.3756_3759del	frameshift	rs80357868	1 (1.8%)	P
	Recurrent P/LP variants				
	c.4675G>C	missense	rs80356988	2 (3.8%)	P/LP
	c.4689C>G	stop gained	rs80357433	2 (3.8%)	P
	c.1303_1309del	frameshift	-	2 (3.8%)	P
*BRCA2*	Recurrent P/LP variants				
	c.7879A>T	missense	rs80359014	4 (10.5%)	P
	c.2808_2811del	frameshift	rs80359351	3 (8%)	P
	c.5238dup	frameshift	rs80359499	3 (8%)	P
	c.2830A>T (*)	stop gained	rs80358533	2 (5%)	P
	c.5681dup	frameshift	rs80359527	2 (5%)	P
	c.9253del/dup	frameshift	rs80359752	2 (5%)	P

**Table 4:** Recurrent P/LP variants in non-BRCA1/2 genes in BC patients and controls.

Gene	c.HGVS	SNP ID	Type	BC Patients, n (%)	Controls, n (%)	ACMG
*ATM*	c.5932G>T	rs587779852	nonsense	5 (0.6%)	1 (0.2%)	P
*BARD1*	c.1690C>T	rs587780021	nonsense	2 (0.2%)	0 (0%)	P
*CHEK2*	c.1100del	rs555607708	frameshift	10 (1.2%)	3 (0.6%)	P
	c.444+1G>A	rs121908698	splice site	6 (0.7%)	1 (0.2%)	P/LP
	c.433C>T	rs137853007	missemse	4 (0.5%)	0 (0%)	P/LP
*FANCC*	c.455dup	rs774170058	frameshift	3 (0.3%)	0 (0%)	P
*MUTYH*	c.1103G>A	rs36053993	missense	2 (0.2%)	2 (0.4%)	P/LP
	c.650G>A	rs140342925	missense	2 (0.2%)	0 (0%)	P/LP
*NBEAL1*	c.3463G>T	rs200689887	nonsense	5 (0.6%)	0 (0%)	LP
*NBN*	c.657_661del	rs587776650	frameshift	6 (0.7%)	3 (0.6%)	P
*XRCC2*	c.96del	rs730882048	frameshift	7 (0.8%)	0 (0%)	LP

**Table 5:** Patients with a combination of two P/LP variants.

Patient ID	Gene	c.HGVS	SNP ID	ACMG
M56	*BRCA1*	c.1303_1309del	--	P
	*BARD1*	c.362C>G	--	LP
M92	*NBN*	c.657_661del	rs587776650	P
	*BLM*	c.1642C>T	rs200389141	P
M107	*BRCA2*	c.4284dup	rs80359439	P
	*MUTYH*	c.1103G>A	rs36053993	P
M191	*NBN*	c.657_661del	rs587776650	P
	*FANCI*	c.3980_3983del	rs1170252316	LP
MR-4	*BRCA2*	c.7879A>T	rs587776650	P
	*NBN*	c.657_661del	rs587776650	P
MR-353	*CHEK2*	c.433C>T	rs137853007	LP
	*ERCC2*	c.1703_1704del	rs587778271	P/LP
MR-370	*BRCA1*	c.4675G>A	rs80356988	P
	*NBN*	c.657_661del	rs587776650	P
MR-404	*BRCA1*	c.4689C>G	rs80357433	P
	*WRN*	c.2665C>T	rs774765029	P

**Table 6:** Clinical features and P/LP variants in patients with PMMTs (BC—breast cancer, TC—thyroid cancer, CRC—colorectal cancer, LC—lung cancer, HL—Hodgkin lymphoma, BTC—biliary tract cancer, non—nonsense, fms—frameshift, mis—missense, spl—splicing).

Patient ID	1st Tumor (Age)	2nd Tumor, (Age)	3rd Tumor (Age)	Gene	c.HGVS	Type	SNP ID	ACMG
M5	CRC (58)	BC (64)		*ATM*	c.4148C>A	Non	rs141087784	P
M11	BC (39)	TC (59)		*ATM*	c.5932G>T	Non	rs587779852	P
M61	CRC (55)	BC (62)		*ATM*	c.5932G>T	Non	rs587779852	P
M214	CRC (67)	BC (68)	OC (68)	*BRCA2*	c.6591_6592del	Fms	rs80359605	P
MR10	BC (33)	LC (33)		*BRCA2*	c.3751dup	Fms	rs397507683	P
M21	TC (46)	BC (47)		*CHEK2*	c.444+1G>A	Spl	rs121908698	P
MR628	HL (18)	BC (34)		*FANCC*	c.455dup	fms	rs774170058	P
M107	BC (48)	BTC (48)		*MUTYH*	c.1103G>A	mis	rs36053993	P
M124	BC (64)	BC (64)	CRC (67)	*RAD52*	c.186+1G>A	Spl	-	LP

## Data Availability

The data are not publicly available due to restrictions; these data contain information that could compromise the privacy of research participants. Requests to access the additional data should be addressed to the following email: s.nikolaev@mknc.ru.

## Supplementary Materials

The following supporting information can be downloaded at: https://www.mdpi.com/article/10.3390/ijms252312640/s1.
