# Supplementary material for: High- and Moderate-Risk Variants Among Breast Cancer Patients and Healthy Donors Enrolled in Multigene Panel Testing in a Population of Central Russia
Source: Int J Mol Sci. 2024 Nov 25;25(23):12640. doi: 10.3390/ijms252312640 (PMC11641773; doi:10.3390/ijms252312640)
Supplement: Supplementary file 1 [file ijms-25-12640-s001.zip › Supplementary/Tables S4, S5, S6, S8.pdf]

Table S4. Association between ER status and the presence of P/LP variants (statistically significant associations are marked in bold).

Gene	Genotype	ER-positive	ER-negative	OR (95% CI)	p-value
ATM	mut	9 (1.2%)	1 (0.5%)		
	wt	581	209	0.31 (0.05 -3.26)	0.2
BARD1	mut	3 (0.5%)	2 (1%)		
	wt	587	208	1.88 (0.31-11.34)	0.5
BRCA1	mut	25 (4.2%)	23 (10.9%)		
	wt	565	189	2.78 (1.54-5.01)	**0.0009**
BRCA2	mut	25 (4.2%)	8 (3.8%)		
	wt	562	202	0.9 (0.40 – 2.02)	0.79
CHEK2	mut	20 (3.4%)	0 (0%)		
	wt	570	210	0.00(0.00-NA)	**0.0004**
FANCC	mut	3 (0.5%)	1 (0.5%)		
	wt	587	209	0.94 (0.10 – 9.05)	0.95
MUTYH	mut	5 (0.8%)	2 (1%)		
	wt	585	208	1.12 (0.22 -5.84)	0.89
NBEAL1	mut	1 (0.2%)	2 (1%)		
	wt	589	208	5.66 (0.51 – 62.79)	0.14
NBN	mut	6 (1%)	0 (0%)		
	wt	584	210	0.00 (0.00 - NA)	0.055
PALB2	mut	4 (0.7%)	1 (0.5%)		
	wt	586	209	0.70 (0.08 – 6.31)	0.74
RAD50, 51C, 54B	mut	7 (1.2%)	2 (1%)		
	wt	583	208	0.80 (0.17 – 3.89)	0.78
SLX4	mut	2 (0.3%)	0 (0%)		
	wt	588	210	0.00 (0.00 - NA)	0.27
XRCC2	mut	4 (0.7%)	2 (1%)		
	wt	586	208	1.4 (0.26 – 7.75)	0.6

Table S5. Association between bilaterality of BC and the presence of P/LP variants (statistically significant associations are marked in bold).

Gene	Genotype	Unilateral BC	Bilateral BC	OR (95% CI)	p-value
ATM	mut	11 (1.3%)	1 (2.6%)		
	wt	812	37	2.0 (0.25 -15.80)	0.4
BARD1	mut	5 (0.6%)	0 (0%)		
	wt	817	38	0.00 (0.00 - NA)	0.76
BRCA1	mut	47 (5.7%)	7 (18.4%)		
	wt	775	31	**3.72 (1.91 – 10.12)**	**0.007**
BRCA2	mut	35 (4.3%)	3 (8.6%)		
	wt	787	35	1.93 (0.56 – 6.57)	0.23
CHEK2	mut	21 (2.5%)	0 (0%)		
	wt	801	38	0.00 (0.00-NA)	0.17
FANCC	mut	4 ((0.5%)	0 (0%)		
	wt	818	38	0.00 (0.00-NA)	0.55
MUTYH	mut	7 (0.8%)	0 (0%)		
	wt	815	38	0.00 (0.00-NA)	0.89
NBEAL1	mut	3 (0.4%)	0 (0%)		
	wt	819	38	0.00 (0.00 - NA)	0.6
NBN	mut	6 (0.7%)	0 (0%)		
	wt	816	38	0.00 (0.00 - NA)	0.055
PALB2	mut	5 (0.6%)	0 (0%)		
	wt	817	38	0.00 (0.00 - NA)	0.5
RAD50	mut	4 (0.5%)	1 (2.6%)		
	wt	818	37	5.53 (0.34 – 22.57)	0.2
TP53	mut	2 (0.2%)	1 (2.6%)		
	wt	820	37	11.1 (0.98 – 125.1)	0.12
SLX4	mut	3 (0.4%)	0 (0%)		
	wt	819	38	0.00 (0.00 - NA)	0.6
XRCC2	mut	7 (0.8%)	0 (0%)		
	wt	815	38	0.00 (0.00-NA)	0.89

Table S6. Association between family history of BC or OC and the presence of P/LP variants (statistically significant associations are marked in bold).

Gene	Genotype	No history	BC/OC history	OR (95% CI)	p-value
ATM	mut	10 (1.5%)	2 (1.1%)		
	wt	656	175	0.83 (0.18 -3.90)	0.81
BARD1	mut	4 (0.6%)	1 (0.6%)		
	wt	662	176	0.94 (0.10 – 8.47)	0.96
BRCA1	mut	28 (4.2%)	24 (13.6%)		
	wt	638	153	3.57 (2.02 – 6.34)	**<0.0001**
BRCA2	mut	28 (4.3%)	10 (5.7%)		
	wt	638	167	1.36 (0.65 – 2.87)	0.42
CHEK2	mut	12 (1.8%)	8 (4.5%)		
	wt	654	169	2.58 (1.04 – 6.41)	**0.05**
FANCC	mut	3 (0.4%)	0 (0%)		
	wt	663	177	0.00 (0.00-NA)	0.23
MUTYH	mut	6 (0.9%)	1 (0.6%)		
	wt	660	176	0.65 (0.07- 5.23)	0.65
NBEAL1	mut	3 (0.4%)	0 (0%)		
	wt	663	177	0.00 (0.00 - NA)	0.23
NBN	mut	6 (0.9%)	0 (0%)		
	wt	660	177	0.00 (0.00 - NA)	0.092
PALB2	mut	4 (0.6%)	1 (0.6%)		
	wt	662	176	0.94 (0.10 – 8.47)	0.96
RAD50	mut	4 (0.6%)	1 (0.6%)		
	wt	662	176	1.08 (0.22 – 5.23)	0.93
SLX4	mut	3 (0.4%)	0 (0%)		
	wt	663	177	0.00 (0.00 - NA)	0.23
XRCC2	mut	4 (0.6%)	1 (0.6%)		
	wt	662	176	0.94 (0.10 – 8.47)	0.96

Table S8. Association between primary multiple tumors and the presence of P/LP variants (statistically significant associations are marked in bold).

Gene	Genotype	Only BC	PMT	OR (95% CI)	p-value
ATM	mut	9 (1.0%)	3 (8.8%)		
	wt	817	31	8.79 (2.26 -34.07)	**0.01**
BARD1	mut	4 (0.5%)	1 (2.9%)		
	wt	822	33	6.23 (0.68 – 57.27)	1.00
BRCA1	mut	54 (6.5%)	0 (0%)		
	wt	772	34	0.00 (0.00-NA)	1.00
BRCA2	mut	35 (4.2%)	3 (8.8%)		
	wt	791	31	2.19 (0.64 – 7.50)	0.26
CHEK2	mut	20 (2.4%)	1 (2.9%)		
	wt	806	33	1.22 (0.16 – 9.38)	0.85
FANCC	mut	3 (0.5%)	1 (2.9%)		
	wt	823	33	8.31 (0.84-82.08)	0.14
MUTYH	mut	6 (0.7%)	0 (0%)		
	wt	820	34	4.14 (0.48 -35.39)	0.89
NBEAL1	mut	3 (0.4%)	0 (0%)		
	wt	823	34	0.00 (0.00 - NA)	1.00
NBN	mut	6 (0.7%)	0 (0%)		
	wt	820	34	0.00 (0.00 - NA)	0.49
PALB2	mut	5 (0.6%)	0 (0%)		
	wt	821	34	0.00 (0.00 - NA)	0.52
RAD50, 51C, 54B	mut	9 (1.1%)	0 (0%)		
	wt	817	34	0.00 (0.00 - NA)	0.39
SLX4	mut	3 (0.4%)	0 (0%)		
	wt	823	34	0.00 (0.00 - NA)	0.62
